# Focus on the Controversial Aspects of ^64^Cu-ATSM in Tumoral Hypoxia Mapping by PET Imaging

**DOI:** 10.3389/fmed.2015.00058

**Published:** 2015-08-24

**Authors:** Mathilde Colombié, Sébastien Gouard, Mathieu Frindel, Aurélien Vidal, Michel Chérel, Françoise Kraeber-Bodéré, Caroline Rousseau, Mickaël Bourgeois

**Affiliations:** ^1^Institut de Cancérologie de l’Ouest, Saint-Herblain, France; ^2^CRCNA, INSERM, Université de Nantes, Nantes, France; ^3^GIP ARRONAX, Saint-Herblain, France; ^4^Service de Médecine Nucléaire – CHU de Nantes, Nantes, France

**Keywords:** cancer, [^64^Cu]-ATSM, hypoxia, positron emission tomography, reactive oxygen species, radiopharmaceutical

## Abstract

Mapping tumor hypoxia is a great challenge in positron emission tomography (PET) imaging as the precise functional information of the biological processes is needed for many effective therapeutic strategies. Tumor hypoxia has been widely reported as a poor prognostic indicator and is often associated with tumor aggressiveness, chemo- and radio-resistance. An accurate diagnosis of hypoxia is a challenge and is crucial for providing accurate treatment for patients’ survival benefits. This challenge has led to the emergence of new and novel PET tracers for the functional and metabolic characterization of tumor hypoxia non-invasively. Among these tracers, copper semicarbazone compound [64Cu]-diacetyl-bis(*N*^4^-methylthiosemicarbazone) (=64Cu-ATSM) has been developed as a tracer for hypoxia imaging. This review focuses on 64Cu-ATSM PET imaging and the concept is presented in two sections. The first section describes its *in vitro* development and pre-clinical testing and particularly its affinity in different cell lines. The second section describes the controversial reports on its specificity for hypoxia imaging. The review concludes that 64Cu-ATSM – more than a hypoxic tracer, exhibits tracer accumulation in tumor, which is linked to the redox potential and reactive oxygen species. The authors concluded that 64Cu-ATSNM is a marker of over-reduced cell state and thus an indirect marker for hypoxia imaging. The affinity of 64Cu-ATSM for over-reduced cells was observed to be a complex phenomenon. And to provide a definitive and convincing mechanism, more *in vivo* studies are needed to prove the diagnostic utility of 64Cu-ATSM.

## Introduction

Tumor hypoxia is a common and important feature of the tumoral microenvironment, and a well-known consequence of an inadequate supply of dioxygen (O_2_) in a wide range of malignant solid tumors. This hypoxic phenomenon is mainly linked to the imbalance between the high rate of cell proliferation potential during the carcinogenesis process compared to the more slower neoangiogenesis. Despite the difference between biochemists (${\text{O}}_{2}^{-}$ limited electron transport in mitochondria) and physiologists (reduced O_2_ availability due to a decreased O_2_ partial pressure) definition, the hypoxia clinical aspect with a limited oxygen delivery to the aerobic neoplastic and stromal cells is frequently observed in various tumor type with very low oxygen levels where the partial pressure in oxygen is <5 versus 40–60 mmHg in normal tissues (1).

The origin of hypoxia regions in human tumors was postulated by Thomlinson and Gray in 1955s with some observation based on the diminution of the oxygen diffusion with the blood supply distance (2). This limited diffusion of oxygen at distance (about 100 μm) of capillary blood vessels is at the origin of a chronic diffusion hypoxia. In parallel, an acute perfusion hypoxia closest to the capillary vessel was observed. This acute event was the consequence of functionally and structurally defective vascular network in tumor (overdilated, hyperpermeable, tortuous, and disrupted), combined with the high-interstitial pressure of the extracellular matrix, which compress the vessel and reduce the blood flow.

From physiopathological aspect, hypoxia is heterogenous in time and in space and not only accounts for tissue necrosis but also a strong impact on tumor biology and has several bad pronostic for patient clinical outcome. In fact, during the tumor malignant growth, hypoxic area is correlated with an increased genetic instability and more aggressive phenotype which conduct to a strongly associated tumor metastasis risk. Likewise, hypoxia causes unequivocally some resistances to cancers treatments. It has been known from many years that hypoxic condition could cause an intrinsic chemoresistance (by different mechanisms like diminution of the drug concentration in relation with the blood vessel distance, loss of sensitivity to p53-mediated apoptosis, or diminution of cell proliferation by metabolic stress) and generate a resistance to killing by ionizing radiation [diminution of the free radical damage on the intracellular reactive oxygen species (ROS) during hypoxia event].

**Figure 1:**
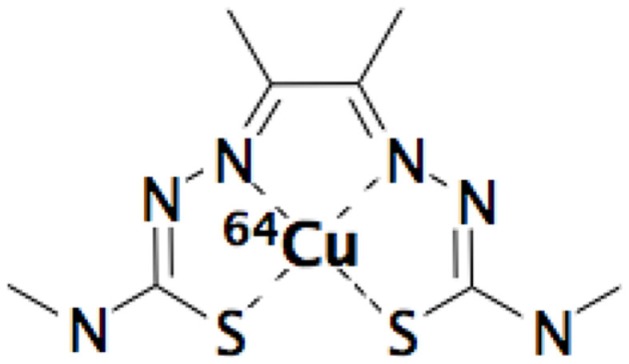
**[^64^Cu]-diacetyl-bis(*N*^4^-methylthiosemicarbazone) (***=***^64^Cu-ATSM)**.

During 1990s, interest in overcoming the problem of the radiation resistance of hypoxic cells in tumors was rekindled by the use of commercially available oxygen electrode (mostly known as “Eppendorf electrode”), which permit to measure oxygen partial pressure levels (pO_2_) in human tumors. However, this method presents some major drawbacks because it is an invasive technique limited to accessible tumors with sufficient size with a risk of tissue disruption and is known to present a large inter-observer variability. In addition to this direct measurement of pO_2_, an other invasive method based on biopsy coupled to cytological coloration with nitroimidazole compounds (like pimonidazole or EF5) or coupled to immunohistochemical analysis of various hypoxia markers proteins [like hypoxia inducing factors-1α (HIF-1α) or carbonic anhydrase IX (CAIX)]. In recent decades, investigations into alternative, non-invasive imaging methods for measuring pO_2_ have been studied and the use of positron emission tomography (PET) has led to a number of promising positron emitters radiopharmaceuticals. Among the different tumor hypoxia PET radiotracers, the innovative [^64^Cu]-diacetyl-bis(*N*^4^-methylthiosemicarbazone) (=[^64^Cu]-ATSM) presented in Figure 1 raises questions about its real target in tumor hypoxia process and presents some controversial aspects.

## Cu-ATSM *In vitro* Results

**Figure 2:**
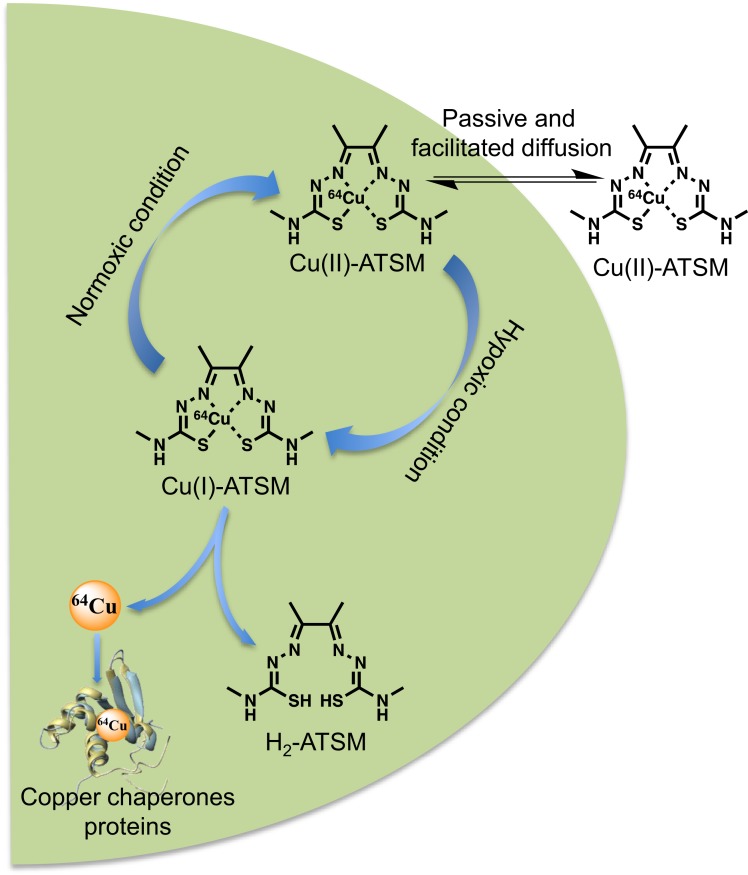
**Overview of the cellular ^64^Cu-ATSM uptake and retention mechanisms**. In hypoxic condition, the Cu(II)-ATSM (oxidation level of copper-64 is +II) is reduced to Cu(I)-ATSM (oxidation level of copper-64 is +I) then the complex became instable and free copper-64 is trapped and accumulate in intracellular copper chaperones proteins.

An ideal hypoxia imaging agent should have a high membrane permeability for easy access to intracellular mitochondria and a low redox potential to confer stability in normal tissue, but it should be able to be reduced by mitochondria with abnormally high electron concentrations in hypoxic cells. After numerous studies on nitroimidazole compounds for their selective accumulation in hypoxic tumors, as well as in ischemic tissue (3, 4), Cu-ATSM a lipophilic molecule, with high membrane permeability and low redox potential, was presented by Fujiyabashi et al. as a possible hypoxia imaging agent in occluded rat heart model (5). Cu-ATSM was reduced by hypoxic but not by normal mitochondria and Cu-ATSM retention was inversely correlated with accumulation of ^201^TI, a relative myocardial blood flow marker. Different schemas were proposed in the literature (6–8) but, to date, there is a consensus that *in vitro* Cu-ATSM undergoes bioreductive trapping under hypoxic conditions. After cellular entry, Cu(II)-ATSM is reduced to an unstable Cu(I)-ATSM species, a process inducing dissociation of the metal complex and subsequent irreversible trapping of Cu(I) within the cellular copper metabolic processes (9). A simplified mechanism of the reaction of Cu(II)-ATSM with cells reported that the lipophilic molecule may be diffuse into the cells by combined passive and facilitated (protein-carrier-mediated) mechanisms with no evidence to support a role for copper-transporter 1 (Ctr1) in accumulation of the compound (10). In hypoxic cells, Cu-ATSM reacted with thiol groups or redox-active proteins with NADH as a required enzymatic cofactor. The reduced, charged form is less lipophilic and retained in the cell, providing opportunity for it to be reoxidized in the normoxic cell (11). Protonation of the reduced form at the N3 and N6 positions will lead to the complex dissociating and the copper will be irreversibly trapped in the hypoxic cell (Figure 2). R could be a thiol, such as glutathione, or a thiol group of a redox-active protein. Cellular trapping of the copper is dependent on oxygen, pH, and NADH (12). Early on, Burgman et al. indicated considerable variation in ^64^Cu accumulation following incubation with ^64^Cu-ATSM among different cell lines (prostate carcinoma, fibrosarcoma, breast adenocarcinoma, and squamous cell carcinoma) with regard to uptake kinetics, maximum accumulation, and their dependence on oxygen conditions (8).

Recently, different authors explored the relationship between Cu-ATSM and some characteristics of cancer cells. First, Liu et al. showed that *in vitro* cells highly expressing multi-drug resistance (MDR1) had significantly decreased Cu-ATSM retention and enhanced efflux. Knockdown of MDR1 expression signifipcantly enhanced the Cu-ATSM retention and decreased the efflux in MDR1-positive cells (13). Then, Yoshii et al. showed that ^64^Cu-ATSM accumulated in rich regions of CD133^+^ cells with characteristics of cancer stem cells. Therefore, ^64^Cu-ATSM could be a potential imaging or therapy agent for rich regions of CD133^+^ cells, associated with cancer stem cells, within tumors (14). The same team was interested in the evaluation of Cu-ATSM as an indicator of intracellular overreduced states in mitochondrial disorders using cell lines with mitochondrial dysfunction, even under normoxia. Study results showed that Cu-ATSM would be a promising marker of intracellular overreduced states for disorders with mitochondrial dysfunction, such as Parkinson’s disease and Alzheimer disease (15).

Still on the fact that Cu-ATSM is not a PET hypoxia marker in all tumors, Vavere et al. focused on the relationship between Cu-ATSM hypoxia and fatty acid synthase (FAS) expression in prostate cancer cell lines (16). The physiological significance of the fatty acid synthesis pathway in prostate cancers is in the harnessing of its oxidizing power for improving redox balance (i.e., lower NADH/NAD^+^ ratios) despite oxygen-limiting (hypoxic) conditions. This team demonstrated that in the FAS tumor line, the lower-than-average redox potential caused reduction and trapping of Cu-ATSM in both hypoxic and normoxic areas (16). Then, Cu-ATSM translation to the hypoxia imaging of prostate cancer may be limited by the overexpression of FAS associated with prostatic malignancies.

## Cu-ATSM *In vivo* Results

The current knowledge on the tumor microenvironment shows a great number of metabolic circumstances and therefore a high variability in hypoxia tumor status with different physiopathological process.

One of the first ^64^Cu-ATSM pre-clinical study in tumor hypoxia imaging has used a mice bearing EMT6 breast carcinoma cell line, which has shown an heterogeneous uptake of the radiotracer (intense uptake was observed in 15–30% of the tumor) supposed to be correlated with the hypoxic area of the tumor (11). Other pre-clinical study in tumor hypoxia imaging has used an epidermoid rabbit tumor, which is known to present a high glycolytic/high hexokinase rate with high anaerobic glycolysis pathway (high lactic acid production and high NADPH ratio) (17). This study showed a major accumulation of ^64^Cu-ATSM around the outer rim of the tumor masses where the histological cell biology showed active, viable, and expected hypoxic cells (18).

More recently, the affinity of ^64^Cu-ATSM for viable and hypoxic cells was confirmed with the comparison of the regional distribution between ^64^Cu-ATSM and other prominent radiopharmaceuticals in tumor metabolic status determination field: ^18^F-MISO, ^18^F-FLT, and ^18^F-FDG. The characterization of the *in vivo* behavior of ^64^Cu-ATSM indicate a very strong correlation with classical hypoxia (^18^F-MISO) and proliferation (^18^F-FLT) PET radiotracer (*r*^2^ = 0.864 and 0.829, respectively) but not correlate with the ^18^F-FDG metabolic PET radiotracer (*r*^2^ = 0.08) (19).

In the same way, a rat graft tumor model of prostate adenocarcinoma and human squamous cell carcinoma showed a good correlation between the uptake of ^18^F-MISO and ^64^Cu-ATSM when imaged at later times after injection (20). Effectively, it appeared that the intratumoral distribution of ^64^Cu-ATSM exhibited a significant evolution between the early (1–2 h after injection) and late (16–20 h after injection) imaging time. An additional experience with direct pO_2_ measurement was broadly consistent with the hypothesis that the spatial distribution of ^18^F-MISO and ^64^Cu-ATSM at later times reflected tumor hypoxia. A similar study indicated that for early images, the distribution of Cu-ATSM was inconsistent with tumor hypoxia and might be more representative of perfusion. Only at later times after Cu-ATSM administration (16–24 h postinjection), the ^18^F-FMISO and ^64^Cu-ATSM images corresponded. Authors did not dispute the potential utility of Cu-ATSM imaging as a tool in clinical management but for the first time, they pointed out that its uptake on hypoxic tumor was unclear (8). In the same way, a more recent study of McCall et al. tried to determine the pharmacokinetic behavior of ^64^Cu-ATSM in combination with microscopic markers of hypoxia. The results of this study confirmed a rapid tumor uptake and retention of ^64^Cu-ATSM (tumor-to-muscle ratio was 4:1 within 20 min after injection) with a strong positive spatial correlation to the highly perfused areas. At late time (18 h post injection), the tumor-to-muscle ratio was 12:1 and there was no spatial correlation with the perfused areas (21).

Furthermore, this time-dependent spatial distribution of ^64^Cu-ATSM seemed to have retention variability in function of the tumor cell line. Briefly, a pre-clinical rat model was used and different tumor lines showed that ^64^Cu-ATSM was a valid PET hypoxia marker (correlation of the autoradiographic distributions with hypoxia markers as EF5, pimonidazole, and CAIX) for adenocarcinoma and glioma tumor cell line but a hypoxia-independent uptake of ^64^Cu-ATSM in fibrosarcoma was observed (22). Cell-dependent distribution and retention kinetics of Cu-ATSM are confirmed and underline the need for proper validation of animal models and PET acquisition protocols before exploration of any new clinical applications (23). This notion has recently been confirmed by Carlin et al., who showed that Cu-ATSM had the highest tumor accumulation and low renal clearance compared to fluorinated nitroimidazoles. However, the lack of correlation between Cu-ATSM distribution and immunohistochemistry hypoxia markers also casted some doubt on the hypoxia selectivity of Cu-ATSM (24). The suggested reason for the low correlation between Cu-ATSM uptake and hypoxic distribution, in some tumors, was the differing redox status of the tumor types. Some of tumors might have a lower than-average redox potential with high concentrations of electron donors caused reduction and trapping of Cu-ATSM in both hypoxic and normoxic areas. Moreover, *in vivo*, in two different tumor types, Hueting et al. demonstrated that the distribution of radiocopper from Cu-ATSM in tumors essentially mirrors Cu-acetate suggesting that copper metabolism might also played a role in the mechanism of selectivity of Cu-ATSM (25). The mechanism of radiolabeled Cu-ATSM accumulation in hypoxic tumor area was currently under investigation but it was well-known than there was a high physiological accumulation of free ^64^Cu in non-target organ and the liver was reported to be the principal dose-limiting organ (26). To reduce the liver absorbed dose, Yoshii et al. showed that the use of a copper chelator like penicillamine could reduce liver absorbed dose (increase of free copper renal clearance) but have no effect on the Cu-ATSM tumor accumulation (27).

In 2008, a clinical use of Cu-ATSM in cancer of the uterine cervix in 10 women permits to obtain high-quality images (high tumor-to-muscle ratio), which correlate with prognosis and patients outcome. Importantly, in this study, the uptake pattern was similar on the images obtained with two different imaging sessions 1–9 days apart, indicating that the microscopic distribution of chronic hypoxia did not change greatly over this interval (28).

## Discussion/Conclusion

^64^Cu-ATSM is a radiopharmaceutical developed for PET imaging and presented as a complex with high membrane permeability and low redox potential, ideal for hypoxia imaging. In theory, low redox potential helped to confer stability in normal tissue and led reduction by mitochondria with abnormally high electron concentrations in hypoxic cells.

During the *in vivo* biodistribution, ^64^Cu-ATSM is known to present a high binding ratio to the serum proteins like albumin (approximately 95% for human, mouse, and rat) (29). The cellular uptake data of ^64^Cu-ATSM suggested by various study show a combined passive diffusion and facilitated (protein-carrier-mediated) penetration mechanisms (10). In particular, organs like liver, the ^64^Cu-ATSM complex is metabolized and free copper released follow its own metabolism with a high fixation on hepatobiliary tractus (30). This metabolic pathway could also occur in tumor cells and could explain the variable time-dependent spatial distribution in hypoxic tumors. From this physiological metabolism of copper, it appears than free copper present a negative impact on the PET image information of ^64^Cu-ATSM. Nevertheless, this impact could be decreased by the co-administration of d-penicillamine, which will permit to accelerate the elimination of free copper without impact on the ^64^Cu-ATSM tumor fixation (27).

^64^Cu-ATSM has been examined in various *in vitro* and *in vivo* pre-clinical models and presented some variability for *in vivo* hypoxia mapping (mainly in term of cell line type and in term of acquisition time after injection). This heterogeneity in ^64^Cu-ATSM uptake led to a complicated interpretation of tumor hypoxia mapping, and there is a need to determine what extent radiotracer distribution is important, defined by perfusion (early time) or by pO_2_ level (late time).

**Figure 3:**
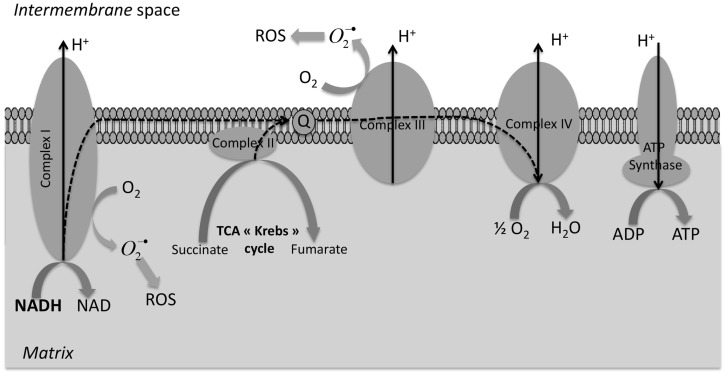
**Overproduction of reductive species (NADPH and NADH) in mitochondria during hypoxia**. Under normoxia, NADH produced during the tricarboxylic acid (TCA) cycle are reduced in NAD^+^ and the respiratory chain drove the electron (dash arrow) to dioxygen with a proton flux on the intermembrane space whose flow back on the mitochondrial matrix by the ATP synthase produced ATP. During hypoxia, the respiratory chain is inhibited and we observe an accumulation of overreduced species (NADH and NADPH) and a diminution (in long-term) of ROS production.

In hypoxia biochemical pathway, the intracellular redox potential becoming progressively more reductive – this agreed with the observation that hypoxia induced a metabolic switch, which led to an increase (Figure 3) in the production of NADH and NADPH (the primary electron donors of the cell). This modification of the NADH/NAD^+^ and NADPH/NADP^+^ ratio in favor of reductive species (confirmed with the use of rotenone, a complex 1-inhibitor in mitochondria electron transport chain) seemed to be at the origin of the Cu-ATSM increased uptake in normoxic cells (15). Indeed, a disturbed electron flow in the electron transport chain, by inhibition of complex 1, caused reduction of Cu(II) to Cu(I) trapped in cells and fourfold higher reduction of Cu-ATSM was observed in normoxic cells treated with rotenone. Yoshii et al. deduced the supposed mechanism of Cu-ATSM retention in hypoxic cells: mitochondrial dysfunction chain (artificial or as result of hypoxia) caused an excess of electrons, and therefore, an over-reduced state in cells. Obata et al. also studied Cu-ATSM retention mechanism (18). They found some contribution of enzymes, in particular, NADH-cytochrome *b*5 reductase and NADPH-cytochrome *p*450 reductase. In addition, those enzymatic reductions were enhanced by induction of hypoxia. Ability in reduction was very sensitive and dependent of electron donors that are NADH or NADPH. They found that Cu(II) reduction depended on presence of NADH and NADPH in cells. More information on hypoxia, Cu-ATSM might appear to provide information on reductive enzyme expression and species in cells. Moreover, literature highlighted an increase of NADH concentration in hypoxic tissue (31). Finally, there was a strong link between hypoxia and redox potential in cells. Yoshii et al. also studied Cu-ATSM based on mitochondrial dysfunction models, including MELAS mitochondrial DNA mutation and cells depleted of mitochondrial DNA; their hypothesis was based on mitochondrial dysfunction and over-reduced state in cells (15). In their study, Cu-ATSM accumulated in cells, which had a strong reducing potential, including normoxic cells, with very strong correlation with rate of NADH and NADPH. Authors suggested that Cu-ATSM could be an agent indicator of over-reduced intracellular state generated by increase of NADH and NADPH levels, including normoxic cells. Cu-ATSM seemed to be more than hypoxia agent, even if redox state and hypoxia were both related and dependent phenomena. This data would explain uptake variations according to cell types (8, 13, 16) and an explication of the low uptake of certain prostate tumor which overexpressed the FAS because this enzyme required a large amount of NADPH as cofactor for function. As previously described, impairment of the respiratory chain generated in a first time excessed electrons, which caused a quickly over-reduced intracellular state, which caused in first time a generation of ROS, increasing oxidative stress and damaging surrounding cells. This overproduction of ROS from mitochondria (32) leads to an oxidative stress in a tumor, which becomes hypoxic (33). In second time, this oxidative stress mediated the shift in oxidative phosphorylation to anaerobic glycolysis (down-regulation of mitochondria mediated by HIF, the major chronic mechanism of hypoxia adaptation in cells) to decrease ROS levels (34).

Finally, ^64^Cu-ATSM seemed to be a marker of the over-reduced cell state and consequently an indirect marker of the hypoxia. In fact, during a severe and chronic hypoxia, the over-reduced cell state conduced to a down-regulation of the mitochondria oxidative phosphorylation and had for consequence a diminution of intracellular ROS. This affinity of Cu-ATSM for over-reduced cell was a complex phenomenon with various possibility of adaptive tumor cells response. To provide a direct answer to radiation oncologists (35) requirement, more studies were needed to determine whether the Cu-ATSM uptake was linked to the low ROS level in cell. Moreover, the first clinical studies has shown a predictive response to traditional cancer therapies in patients with rectal (36), lung (37), and uterine cervix cancer (28, 38) where Cu-ATSM uptake was of worse prognosis.

## Conflict of Interest Statement

The authors declare that the research was conducted in the absence of any commercial or financial relationships that could be construed as a potential conflict of interest.

## References

[1] Brown JM Wilson WR Exploiting tumour hypoxia in cancer treatment. Nat Rev Cancer (2004) 4:437–47. 10.1038/nrc1367 15170446

[2] Thomlinson RH Gray LH The histological structure of some human lung cancers and the possible implications for radiotherapy. Br J Cancer (1955) 9:539–49. 10.1038/bjc.1955.55 13304213 PMC2073776

[3] Chapman JD Baer K Lee J. Characteristics of the metabolism-induced binding of misonidazole to hypoxic mammalian cells. Cancer Res (1983) 43:1523–8. 6831401

[4] Hoffman JM Rasey JS Spence AM Shaw DW Krohn KA. Binding of the hypoxia tracer [3H]misonidazole in cerebral ischemia. Stroke (1987) 18:168–76. 10.1161/01.STR.18.1.168 3810750

[5] Fujibayashi Y Taniuchi H Yonekura Y Ohtani H Konishi J Yokoyama A. Copper-62-ATSM: a new hypoxia imaging agent with high membrane permeability and low redox potential. J Nucl Med (1997) 38:1155–60. 9225812

[6] Dearling JLJ Lewis JS Mullen GED Welch MJ Blower PJ. Copper bis (thiosemicarbazone) complexes as hypoxia imaging agents: structure-activity relationships. J Biol Inorg Chem (2002) 7:249–59. 10.1007/s007750100291 11935349

[7] Maurer RI Blower PJ Dilworth JR Reynolds CA Zheng Y Mullen GED. Studies on the mechanism of hypoxic selectivity in copper bis (thiosemicarbazone) radiopharmaceuticals. J Med Chem (2002) 45:1420–31. 10.1021/jm0104217 11906283

[8] Burgman P O’Donoghue JA Lewis JS Welch MJ Humm JL Ling CC. Cell line-dependent differences in uptake and retention of the hypoxia-selective nuclear imaging agent Cu-ATSM. Nucl Med Biol (2005) 32:623–30. 10.1016/j.nucmedbio.2005.05.003 16026709

[9] Obata A Yoshimi E Waki A Lewis JS Oyama N Welch MJ Retention mechanism of hypoxia selective nuclear imaging/radiotherapeutic agent cu-diacetyl-bis(N4-methylthiosemicarbazone) (Cu-ATSM) in tumor cells. Ann Nucl Med (2001) 15:499–504. 10.1007/BF02988502 11831397

[10] Price KA Crouch PJ Volitakis I Paterson BM Lim S Donnelly PS Mechanisms controlling the cellular accumulation of copper bis(thiosemicarbazonato) complexes. Inorg Chem (2011) 50:9594–605. 10.1021/ic201334q 21882803

[11] Lewis JS McCarthy DW McCarthy TJ Fujibayashi Y Welch MJ. Evaluation of 64Cu-ATSM in vitro and in vivo in a hypoxic tumor model. J Nucl Med (1999) 40:177–83. 9935074

[12] Dearling JLJ Packard AB. Some thoughts on the mechanism of cellular trapping of Cu(II)-ATSM. Nucl Med Biol (2010) 37:237–43. 10.1016/j.nucmedbio.2009.11.004 20346863

[13] Liu J Hajibeigi A Ren G Lin M Siyambalapitiyage W Liu Z Retention of the radiotracers 64Cu-ATSM and 64Cu-PTSM in human and murine tumors is influenced by MDR1 protein expression. J Nucl Med (2009) 50:1332–9. 10.2967/jnumed.109.061879 19617332

[14] Yoshii Y Furukawa T Kiyono Y Watanabe R Mori T Yoshii H Internal radiotherapy with copper-64-diacetyl-bis (N4-methylthiosemicarbazone) reduces CD133+ highly tumorigenic cells and metastatic ability of mouse colon carcinoma. Nucl Med Biol (2011) 38:151–7. 10.1016/j.nucmedbio.2010.08.009 21315269

[15] Yoshii Y Yoneda M Ikawa M Furukawa T Kiyono Y Mori T Radiolabeled Cu-ATSM as a novel indicator of overreduced intracellular state due to mitochondrial dysfunction: studies with mitochondrial DNA-less ρ0 cells and cybrids carrying MELAS mitochondrial DNA mutation. Nucl Med Biol (2012) 39:177–85. 10.1016/j.nucmedbio.2011.08.008 22033022

[16] Vavere AL Lewis JS. Examining the relationship between Cu-ATSM hypoxia selectivity and fatty acid synthase expression in human prostate cancer cell lines. Nucl Med Biol (2008) 35:273–9. 10.1016/j.nucmedbio.2007.11.012 18355682 PMC3872988

[17] Ko YH Pedersen PL Geschwind JF. Glucose catabolism in the rabbit VX2 tumor model for liver cancer: characterization and targeting hexokinase. Cancer Lett (2001) 173:83–91. 10.1016/S0304-3835(01)00667-X 11578813

[18] Obata A Yoshimoto M Kasamatsu S Naiki H Takamatsu S Kashikura K Intra-tumoral distribution of 64Cu-ATSM: a comparison study with FDG. Nucl Med Biol (2003) 30:529–34. 10.1016/S0969-8051(03)00047-7 12831991

[19] Dence CS Ponde DE Welch MJ Lewis JS. Autoradiographic and small-animal PET comparisons between (18)F-FMISO, (18)F-FDG, (18)F-FLT and the hypoxic selective (64)Cu-ATSM in a rodent model of cancer. Nucl Med Biol (2008) 35:713–20. 10.1016/j.nucmedbio.2008.06.001 18678357 PMC2661147

[20] O’Donoghue JA Zanzonico P Pugachev A Wen B Smith-Jones P Cai S Assessment of regional tumor hypoxia using 18F-fluoromisonidazole and 64Cu(II)-diacetyl-bis(N4-methylthiosemicarbazone) positron emission tomography: comparative study featuring microPET imaging, Po2 probe measurement, autoradiography, and fluorescent microscopy in the R3327-AT and FaDu rat tumor models. Int J Radiat Oncol Biol Phys (2005) 61:1493–502. 10.1016/j.ijrobp.2004.12.057 15817355

[21] McCall KC Humm JL Bartlett R Reese M Carlin S. Copper-64-diacetyl-bis(N(4)-methylthiosemicarbazone) pharmacokinetics in FaDu xenograft tumors and correlation with microscopic markers of hypoxia. Int J Radiat Oncol Biol Phys (2012) 84:e393–9. 10.1016/j.ijrobp.2012.05.005 22727887 PMC3522091

[22] Yuan H Schroeder T Bowsher JE Hedlund LW Wong T Dewhirst MW. Intertumoral differences in hypoxia selectivity of the PET imaging agent 64Cu(II)-diacetyl-bis(N4-methylthiosemicarbazone). J Nucl Med (2006) 47: 989–98. 16741309

[23] Valtorta S Belloli S Sanvito F Masiello V Di Grigoli G Monterisi C Comparison of 18F-fluoroazomycin-arabinofuranoside and 64Cu-diacetyl-bis(N4-methylthiosemicarbazone) in preclinical models of cancer. J Nucl Med (2013) 54:1106–12. 10.2967/jnumed.112.111120 23699667

[24] Carlin S Zhang H Reese M Ramos NN Chen Q Ricketts S-A. A comparison of the imaging characteristics and microregional distribution of 4 hypoxia PET tracers. J Nucl Med (2014) 55:515–21. 10.2967/jnumed.113.126615 24491409 PMC4232233

[25] Hueting R Kersemans V Cornelissen B Tredwell M Hussien K Christlieb M A comparison of the behavior of (64)Cu-acetate and (64)Cu-ATSM in vitro and in vivo. J Nucl Med (2014) 55:128–34. 10.2967/jnumed.113.119917 24337603

[26] Laforest R Dehdashti F Lewis JS Schwarz SW. Dosimetry of 60/61/62/64Cu-ATSM: a hypoxia imaging agent for PET. Eur J Nucl Med Mol Imaging (2005) 32:764–70. 10.1007/s00259-004-1756-x 15785955

[27] Yoshii Y Matsumoto H Yoshimoto M Furukawa T Morokoshi Y Sogawa C Controlled administration of penicillamine reduces radiation exposure in critical organs during 64Cu-ATSM internal radiotherapy: a novel strategy for liver protection. PLoS One (2014) 9:e86996. 10.1371/journal.pone.0086996 24466309 PMC3899369

[28] Lewis JS Laforest R Dehdashti F Grigsby PW Welch MJ Siegel BA. An imaging comparison of 64Cu-ATSM and 60Cu-ATSM in cancer of the uterine cervix. J Nucl Med (2008) 49:1177–82. 10.2967/jnumed.108.051326 18552145 PMC4412029

[29] Basken NE Green MA. Cu(II) bis(thiosemicarbazone) radiopharmaceutical binding to serum albumin: further definition of species dependence and associated substituent effects. Nucl Med Biol (2009) 36:495–504. 10.1016/j.nucmedbio.2009.02.006 19520290 PMC2736129

[30] Wang Y Hodgkinson V Zhu S Weisman GA Petris MJ. Advances in the understanding of mammalian copper transporters. Adv Nutr (2011) 2:129–37. 10.3945/an.110.000273 22332042 PMC3065767

[31] Barlow CH Harken AH Chance B. Evaluation of cardiac ischemia by NADH fluroescence photography. Ann Surg (1977) 186:737–40. 10.1097/00000658-197712000-00013 203234 PMC1396508

[32] Indo HP Davidson M Yen H-C Suenaga S Tomita K Nishii T Evidence of ROS generation by mitochondria in cells with impaired electron transport chain and mitochondrial DNA damage. Mitochondrion (2007) 7:106–18. 10.1016/j.mito.2006.11.026 17307400

[33] Dewhirst MW Cao Y Moeller B. Cycling hypoxia and free radicals regulate angiogenesis and radiotherapy response. Nat Rev Cancer (2008) 8:425–37. 10.1038/nrc2397 18500244 PMC3943205

[34] Solaini G Baracca A Lenaz G Sgarbi G. Hypoxia and mitochondrial oxidative metabolism. Biochim Biophys Acta (2010) 1797:1171–7. 10.1016/j.bbabio.2010.02.011 20153717

[35] Clausen MM Hansen AE Lundemann M Hollensen C Pommer T Munck AF Dose painting based on tumor uptake of Cu-ATSM and FDG: a comparative study. Radiat Oncol (2014) 9:228. 10.1186/s13014-014-0228-0 25319766 PMC4203925

[36] Dietz DW Dehdashti F Grigsby PW Malyapa RS Myerson RJ Picus J Tumor hypoxia detected by positron emission tomography with 60Cu-ATSM as a predictor of response and survival in patients undergoing neoadjuvant chemoradiotherapy for rectal carcinoma: a pilot study. Dis Colon Rectum (2008) 51:1641–8. 10.1007/s10350-008-9420-3 18682881 PMC4962601

[37] Dehdashti F Mintun MA Lewis JS Bradley J Govindan R Laforest R In vivo assessment of tumor hypoxia in lung cancer with 60Cu-ATSM. Eur J Nucl Med Mol Imaging (2003) 30:844–50. 10.1007/s00259-003-1130-4 12692685

[38] Dehdashti F Grigsby PW Lewis JS Laforest R Siegel BA Welch MJ. Assessing tumor hypoxia in cervical cancer by PET with 60Cu-labeled diacetyl-bis(N4-methylthiosemicarbazone). J Nucl Med (2008) 49:201–5. 10.2967/jnumed.107.048520 18199612

